# Association between gut microbiota and post-stroke depression in Chinese population: A meta-analysis

**DOI:** 10.1016/j.heliyon.2022.e12605

**Published:** 2022-12-22

**Authors:** Fang Luo, Chengbing Fang

**Affiliations:** Department of Rehabilitation, Tongde Hospital of Zhejiang Province, Hangzhou, Zhejiang 310000, China

**Keywords:** Post-stroke depression, Gut microbiota, Meta-analysis, Alpha diversity, Phylum

## Abstract

**Background:**

Post-stroke depression (PSD) is a common neuropsychological complication after a stroke with a range of poor outcomes. Evidence of gut microbiota disorder for PSD has recently accumulated. This study aimed to systematically evaluate the association between PSD and gut microbiota.

**Methods:**

We searched PubMed, Web of Science, Embase, and VIP, CNKI, Wangfang without language restrictions for eligible studies and performed a meta-analysis and systematic review to assess the pooled differences in gut microbiota compositions between PSD and healthy individuals.

**Results:**

We included nine eligible studies reporting the differences in the intestinal microbiome between PSD and healthy control. The pooled results demonstrated that the sequencing depth index (Good's coverage), richness indexes (Chao1 and ACE), evenness, and alpha diversity (Shannon and Simpson) were not significantly changed in PSD patients as compared to healthy controls. The observed species (operational taxonomic unit, OUT) in PSD was significantly higher than that in healthy individuals (SMD, 1.86, 95%CI: 1.47 to 2.25). Furthermore, we observed significant differences between PSD and healthy individuals at the phylum level. The pooled estimation of relative abundance of *Proteobacteria* (SMD, 0.37, 95%CI: 0.19 to 0.55), *Bacteroidetes* (SMD, 1.87, 95%CI: 1.25 to 2.48), and *Fusobacteria* (SMD, 1.06, 95%CI: 0.76 to 1.37) in patients with PSD significantly was increased as compared to controls, while the pooled relative abundance of *Firmicutes* (SMD, -0.84, 95%CI: -1.21 to -0.47) was significantly decreased in PSD as compared to healthy controls. Moreover, significant differences in intestinal microbiota were observed between PSD patients and healthy controls at the family and genus levels.

**Conclusions:**

This meta-analysis indicates a significant alteration of observed species and microbiota composition at the phylum, family and genus levels in PSD as compared to healthy individuals.

## 1 Introduction

Stroke is one of the most common and serious neurological diseases and the leading cause of long-term disability in the world. Post-stroke depression (PSD) is a frequent and burdensome neuropsychological complication after a stroke, which often presents with poor outcomes and high mortality rates [1]. The prevalence rate of PSD ranges from 18% to 33% among all stroke survivors [2, 3, 4, 5]. The major symptoms of PSD include melancholia, dysphoria, and vegetative signs such as sleep disorders, reduced libido, and energy level, which results in a poor quality of life [6, 7, 8]. It was proposed that multiple factors including psychosocial distress, alteration of monoamine neurotransmitter, etc. were involved in the pathogenesis of PSD [9]. Current treatment for PSD is essentially pharmacological due to the unclear efficacy of individual psychotherapy [10]. At present, little is known about the specific pathophysiology of PSD, which severely limits the development of PSD therapy.

The intestinal microbiome is the major microbial community that settles in the human body and substantial evidence suggested the crucial role of the intestinal microbiome in stroke onset. Trimethylamine N-oxide (TMAO) is a waste product of gut microbes and was associated with the development of stroke [10]. A recent study demonstrated that serum TMAO level was positively correlated with depressive symptoms [11]. Conversely, stroke can lead to dysbiosis of the gut microbiota and epithelial barrier integrity [12]. Meanwhile, accumulating evidence has also revealed that an altered gut microbiome was implicated in depression [13, 14, 15]. Therefore, recent studies attempted to explore the specific gut microbiome in patients with PSD [16, 17]. The microbiota-gut-brain axis forms a biochemical signal pathway that coordinates communication and interaction between the enteric and central nervous systems, which eventually influence cognitive and emotional function [18]. However, due to the small sample size, different detection methods and analysis pipeline, the results of most studies have large heterogeneity.

In this study, we conducted a meta-analysis of all available studies comparing the gut microbiota in PSD and healthy control. The eligible studies were enrolled from six public databases without language restriction, and relevant data were extracted directly or indirectly from the articles. Finally, we evaluated the pooled differences in intestinal microbiome compositions between PSD patients and controls.

## 2 Materials and methods

### 2.1 Search strategy

For eligible studies collection, we systematically and comprehensively searched in PubMed, Web of Science, Embase, Cochrane databases, Wangfang, VIP, and CNKI up to July 2022 using the following search keywords: “stroke” and “depression” and (“gut” or “intestinal”) and (“microbiota” or “microbiome” or “microorganisms”) without language restriction. This meta-analysis was conducted on the basis of the Preferred Reporting Items for Systematic Reviews and Meta-analyses (PRISMA) criteria.

### 2.2 Inclusion and exclusion criteria

Eligible studies were identified according to the following criteria: 1) comparing the gut microbiota in PSD and healthy controls; 2) providing sufficient data for pooled differences analysis; 3) providing available full text. The exclusion criteria were as follows: review, animal studies, and conference abstract.

### 2.3 Data extraction and quality assessment

The following information for each eligible study includes the first authors’ name, date of publication, country, sample size, detection method, richness and diversity indexes of 16S rRNA-sequencing data, and relative abundance of gut microbiota. Two researchers independently extracted and assessed the data, and any disagreements were resolved by consensus between the reviewers. The methodological quality of included articles was assessed by the selection, comparability, and outcome information of each study according to the Cochrane Non-Randomized Studies Methods Group recommendations [19].

### 2.4 Statistical analysis

All statistical analysis was performed using the STATA SE 15 software. The average and 95%CI of the sequencing depth index (Good's coverage), richness indexes (observed species/OTU, Chao1, ACE, diversity indexes (Shannon and Simpson), and the relative abundance of gut microbiota were extracted and calculated. We evaluate the differences in gut microbiota compositions using the standardized mean differences (SMD) between the PSD and healthy controls, while a positive SMD indicates a higher level in PSD patients than in healthy controls. Conversely, a negative SMD indicates a lower level in PSD patients. The heterogeneity among included research was examined using the *I*^2^ statistic, and a fixed-effect model was used when *I*^2^ > 50%, otherwise, the random-effect model was applied.

## 3 Results

### 3.1 Characteristics of eligible studies

**Figure 1:**
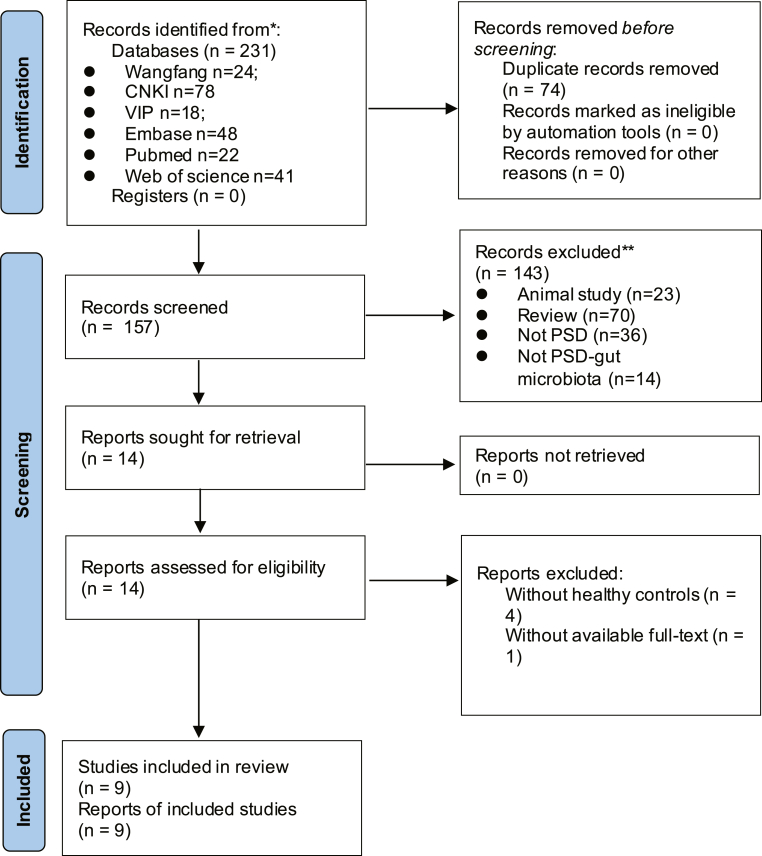
Flow chart of the search strategy and study selection progress.

The flow chart of the literature search and selection progress was shown in Figure 1. A total of 231 relevant articles were retrieved from the six databases, with 74 duplicated research. According to the content of titles and abstracts, 143 articles were discarded due to their not meeting the eligibility criteria. Then, five studies of the remaining 14 articles were eliminated due to the lack of healthy controls and available full text. Finally, nine eligible articles were included in the subsequent meta-analysis [16, 17, 20, 21, 22, 23, 24, 25, 26]. Totally, 826 individuals with 419 patients with PSD and 407 healthy controls were included. Detailed information on the eligible articles were illustrated in Table 1. The nine included research were all performed in China from 7 provinces, and seven of them were published in Chinese. The gut microbiota of eight eligible studies was detected using pyrosequencing or high-throughput sequencing of the region of V3, V3–V4, or the full length of the 16S rRNA gene. Specifically, Yi Kang et al. only detected and reported three components in gut microbiota using ATB-expression semi-automatic microbial detection system [16]. The assessment of the methodological quality found that six studies [16, 17, 20, 21, 22, 23] had a good methodological quality and the rest [24, 25, 26] had a fair quality.

**Table 1:** Technical details of the studies included in the meta-analysis.

first author	Province	Male/Female	Age	BMI	region	Country	Year	PSD/CTR	16S region	Seq. Tech.
Xudong Guo	Henan	36/32^b^	56.2 ± 2.6^b^	NA	North	China	2022	68/68	V3	Barcoded 454
Wentao Fan	Shanxi	18/14^b^	40–65^b^	NA	North	China	2022	32/30	V3	Barcoded 454
Yamei Li	Sichuan	7/5^b^	60.75 ± 12.45^b^	25.20 ± 1.13^b^	South	China	2016	12/12	Full length	PacBio SMR
Guangshun Han	Guangxi	21/15^b^	57.47 ± 7.366^b^	21.19 ± 3.371^b^	South	China	2021	36/30	V3–V4	Illumina Miseq/HiSeq2500
Xinyue Sun	Beijing	15/18^b^	72.20 ± 13.6^b^	NA	North	China	2019	33/10	V3–V4	Illumina Miseq/HiSeq2500
Yanhong Li	Henan	41/35^b^	52. 28 ± 2. 35^b^	NA	North	China	2021	76/76	V3	Barcoded 454
Xuecan Zuo	Henan	36/28^b^	52.3 ± 8.4^b^	NA	North	China	2019	64/60	V3	Barcoded 454
Yi Ling	Zhejiang	17/24^b^	69.63 ± 9.35^b^	25.14 ± 3.62^b^	South	China	2020	41/25	V3–V4	MiSeq Benchtop Sequencer
Yi Kang	Chongqing	85/78^a^	55.91 ± 8.61^a^	NA	South	China	2021	67/96	NA	ATB-expression semi-automatic microbial detection system

Abbreviations: PSD: post-stroke depression, CTR: control; Seq. Tech.: sequencing technology,^a^ represent the data of whole cohorts of PSD and CTR, ^b^ represented the data in the PSD cohort.

### 3.2 General characteristics of 16S rRNA sequencing

To evaluate the pooled differences in general characteristics of high throughout-sequencing between PSD and healthy individuals, we first performed a meta-analysis on multiple relevant indexes, including the sequencing depth (Good's coverage), richness (observed species/OTU, Chao1, ACE), evenness, and alpha diversity (Shannon and Simpson). Seven studies provided available data on general characteristics and were included in the quantitative analysis. The detail information on the meta-analysis were shown in Table 2.

**Table 2:** Meta-analysis of general characteristics of 16s RNA sequencing of PSD.

Terns	Number of studies	Participants	*I*^2^	*p*	Effect [95%CI]	*z*	*p*
Good's Coverage	4	378	89.0	<0.001	-0.057 (-0.701,0.586)	-0.175	0.860
OUT	3	322	52.5	0.122	1.862 (1.472,2.252)	9.357	<0.001
Evenness	3	322	62.4	0.070	-0.15 (-0.52,0.22)	-0.782	0.434
ACE	6	445	92.2	<0.001	0.294 (-0.459,1.048)	0.766	0.444
MFR>1	5	402	90.0	<0.001	0.569 (-0.120,1.258)	1.168	0.106
MFR<1	1	43	NA	NA	-1.123 (-1.871,-0.375)	-2.943	0.003
Age<60	3	316	93.4	<0.001	0.714 (-0.235,1.664)	1.475	0.140
Age>60	2	67	39.6	0.198	-0.778 (-1.487,-0.070)	-2.154	0.031
Age = NA	1	62	NA	NA	0.940 (0.414,1.465)	3.503	<0.001
Region = North	4	365	91.3	<0.001	0.640 (-0.165,1.445)	1.558	0.119
Region = South	2	80	0	0.972	-0.412 (-0.856,0.032)	-1.818	0.069
Chao I	5	389	97.2	<0.001	0.441 (-1.110,1.992)	0.557	0.578
MFR>1	4	346	95.7	<0.001	1.060 (-0.268,2.389)	1.564	0.118
MFR<1	1	43	NA	NA	-1.972 (-1.110,1.992)	-4.680	<0.001
Age<60	2	260	76.4	0.040	2.269 (1.624,2.915)	6.889	<0.001
Age>60	2	67	0	0.951	-1.957 (-2.590,-1.324)	-6.063	<0.001
Age = NA	1	62	NA	NA	1.329 (0.777,1.881)	4.719	<0.001
Region = North	3	365	96.8	<0.001	1.014 (-0.477,2.506)	1.333	0.183
Region = South	1	24	NA	NA	-1.933 (-2.917,-0.950)	-3.854	<0.001
Shannon	6	487	97.5	<0.001	0.757 (-0.615,2.128)	1.082	0.279
MFR>1	4	378	98.3	<0.001	1.529 (-0.382,3.440)	1.568	0.117
MFR<1	2	109	89.0	0.003	-0.763 (-2.156,0.630)	-1.073	0.283
Age<60	3	316	98.8	<0.001	1.877 (-0.920,4.674)	1.315	0.188
Age>60	2	109	89.0	0.003	-0.763 (-2.156,0.630)	-1.073	0.283
Age = NA	1	62	NA	NA	0.532 (0.025,1.040)	2.058	0.040
Region = North	4	365	98.4	<0.001	1.222 (-0.615,2.128)	1.080	0.280
Region = South	2	122	0	0.737	-0.143 (-0.504,0.219)	-0.773	0.439
Simpion	6	455	89.5	<0.001	0.404 (-0.227,1.034)	1.256	0.209
MFR>1	4	109	91.5	<0.001	0.301 (-0.499,1.100)	0.737	0.461
MFR<1	2	346	91.5	0.001	0.646 (-0.940,1.034)	0.798	0.425
Age<60	2	260	94.5	<0.001	0.841 (-0.262,1.943)	1.494	0.135
Age>60	3	133	85.7	0.001	0.318 (-0.227,1.034)	0.593	0.553
Age = NA	1	62	NA	NA	-0.267 (-0.767,0.234)	-1.045	0.296
Region = North	4	365	91.7	<0.001	0.704 (-0.098,1.507)	1.720	0.086
Region = South	2	90	0	0.654	-0.195 (-0.619,0.229)	-0.903	0.367

Abbreviations: PSD:post-stroke depression, OUT: operational taxonomic unit, MFR: male/female ratio.

Four studies reported the Good's coverage index of 16S rRNA-sequencing results in PSD (n = 190) and healthy persons (n = 188). The results revealed an insignificant pooled estimation of Good's coverage index between patients and controls (SMD, -0.057, 95%CI: -0.701 to 0.586, p = 0.860) under the random-effect model due to high heterogeneity (*I*^2^ = 89%). The observed species/OTU were reported 3 articles (164 PSD vs 158 controls), and the pooled analysis revealed a significantly higher observed species/OTU in PSD vs controls (SMD, 1.86, 95%CI: 1.47 to 2.25, *p* = 0.000) and a high heterogeneity (*I*^2^ = 52.5%) among studies. Five studies provided data on Chao1 in PSD (n = 209) and controls (n = 180). The pooled estimate demonstrated an insignificant difference between PSD and controls (SMD, 0.44, 95%CI: -1.11 to 1.99, *p* = 0.578). Meanwhile, six studies with 235 PSD and 210 healthy controls reported ACE data, and the results found no significant difference between PSD and healthy individuals (SMD, 0.29, 95%CI: -0.46 to 1.05, *p* = 0.444). Regarding diversity, six studies provided the Shannon index in 264 PSD patients and 233 controls. Owing to the high heterogeneity (*I*^2^ = 97.5), the pooled effects were evaluated using a random-effect model and showed an insignificant difference between PSD and controls (SMD, 0.76, 95%CI: -0.61 to 2.13, *p* = 0.279). Similarly, the pooled effects of the Simpson index from six studies were also assessed using a random-effects model, with no significant difference observed (SMD, 0.40, 95%CI: -0.23 to 1.03, *p* = 0.209). Finally, three studies with 164 PSD and 158 controls provided sufficient data for the pooled estimate of evenness, which also revealed an insignificant SMD in evenness between groups (*p* = 0.434). Stratification analysis was performed according to limited data of region, male/female ratio (MFR), and average value of age. It was found that age had a significant impact on ACE and Chao I.

### 3.3 Differences in the microbial composition at the phylum level

**Figure 2:**
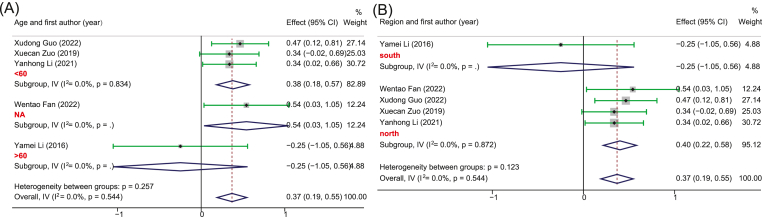
Forest plot of the percentages of *Bacteroidetes* comparing people with PSD to healthy controls. Forest plot of the percentages of *Bacteroidetes* secrified by (A) age and (B) region.

**Figure 3:**
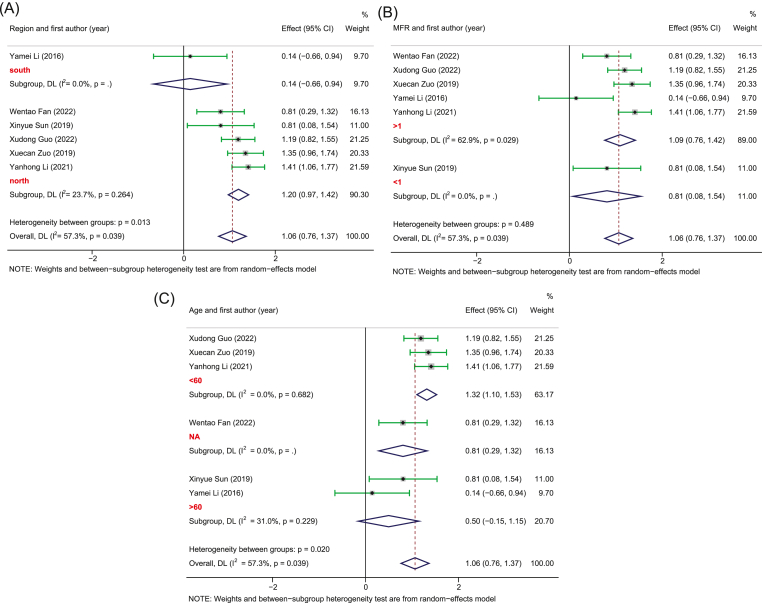
Forest plot of the percentages of *Proteobacteria* comparing people with PSD to healthy controls. Forest plot of the percentages of *Proteobacteria* secrified by (A) region, (B) MFR, and (B) age.

**Figure 4:**
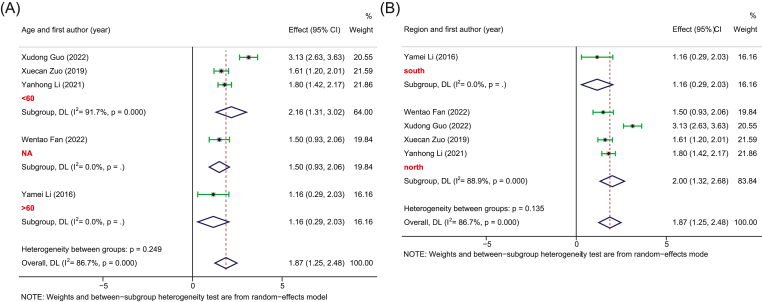
Forest plot of the percentages of *Fusobacteria* comparing people with PSD to healthy controls. Forest plot of the percentages of *Fusobacteria* secrified by (A) age and (B) region.

**Figure 5:**
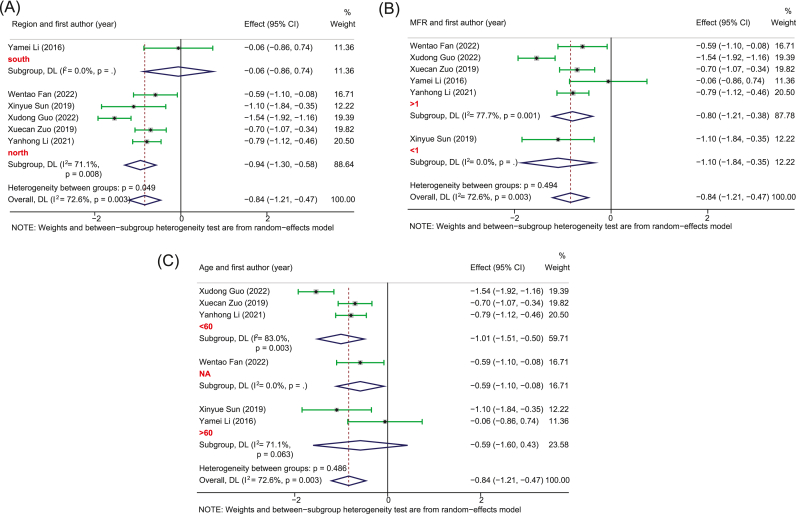
Forest plot of the percentages of *Firmicutes* comparing people with PSD to healthy controls. Forest plot of the percentages of *Firmicutes* secrified by (A) region, (B) MFR, and (B) age.

Five studies with 252 PSD and 246 healthy controls provided sufficient data for the pooled estimate of four dominant bacterial phyla from the gut. The results showed significant differences in bacterial phyla between PSD and controls. Specifically, in terms of *Bacteroidetes*, no heterogeneity was observed and the pooled analysis found a significantly increased relative abundance in PSD (SMD, 0.37, 95%CI: 0.19 to 0.55, *p* = 0.000) as compared to controls (Figure 2). Meanwhile, due to the high heterogeneity, the random-effect remodel was adopted to evaluate the pooled differences in the relative abundance of *Proteobacteria* and *Fusobacteria* between PSD and controls. As a result, we observed a significantly increased relative abundance in PSD as compared to healthy individuals for both *Proteobacteria* (SMD = 1.06, 95%CI: 0.76–1.37, *p* = 0.000) and *Fusobacteria* (SMD = 1.87, 95%CI: 1.25–2.48, *p* = 0.000) (Figures 3 and 4). Stratification analysis demonstrated that PSD cohorts with an average age less than 60, MFR greater than 1, or from the northern area of China had a significantly higher content of *Bacteroidetes*, *Proteobacteria*, and *Fusobacteria* as compared to healthy controls. Conversely, the results demonstrated that PSD patients had a significantly lower content of *Firmicutes* as compared to healthy controls (SMD = -0.84, 95%CI: -1.21 - -0.47, *p* = 0.000) (Figure 5). Meanwhile, stratification analysis revealed that PSD cohorts with an average age less than 60, MFR greater than 1, or from the northern area of China had a significantly lower content of *Firmicutes* as compared to healthy controls.

### 3.4 Differences in the microbial composition at the family and genus level

In order to more comprehensively and clearly describe the characteristics of the intestinal flora of PSD patients, we further conducted a meta-analysis at the family and generic levels. Table 3 illustrated the results of meta-analytic results of 9 families and 8 genera. It was observed that PSD patients had a dramatically lower content of five families, including *Bacteroidaceae*, *Lachnospiraceae*, *Erysipelotrichaceae*, *Prevotellaceae*, and *Ruminococcaceae*. Meanwhile, the intestinal contents of the other 4 families (*Acidaminococcaceae*, *Fusobacteriaceae*, *Enterobacteriaceae*, *Rikenellaceae*) in PSD patients were significantly elevated as compared to the healthy controls. At the genus level, the meta-analysis showed that two genera including *Prevotella* and *Ruminococcus* presented with a lower intestinal content in the intestine of PSD patients. Meanwhile, PSD patients had a significantly higher content of four genera, including *Faecalibacterium*, *Escherichia-Shigella*, *Megamonas*, and *Bacillus*, as compared to the healthy controls. Insignificant differences in the intestinal content of *Roseburia* and *Blautia* were not observed between the PSD patients and healthy controls.

**Table 3:** Meta-analysis of gut microbiota of PSD at the family and genus levels.

Gut microbiota	Number of studies	Participants	*I*^2^	*p*	Effect [95%CI]	*z*	*p*
**Family**							
*Acidaminococcaceae*	4	474	98.7	<0.001	9.551 (6.307,12.796)	5.770	<0.001
*Fusobacteriaceae*	4	474	96.7	<0.001	3.434 (1.853,5.014)	4.259	<0.001
***Bacteroidaceae***	**3**	**412**	**0**	**0.897**	**-0.253 (-0.447,-0.059)**	**-2.553**	**0.011**
***Lachnospiraceae***	**4**	**474**	**0**	**0.971**	**-0.456 (-0.638,-0.273)**	**-4.895**	**<0.001**
*Enterobacteriaceae*	4	474	98.9	<0.001	5.174 (1.731,8.617)	2.945	0.003
***Erysipelotrichaceae***	**4**	**474**	**98.6**	**<0.001**	**-4.306 (-6.729,-1.883)**	**-3.484**	**<0.001**
***Prevotellaceae***	**4**	**474**	**0**	**0.845**	**-0.641 (-0.826,-0.456)**	**-6.796**	**<0.001**
*Rikenellaceae*	4	474	98.3	<0.001	7.599 (4.544,10.653)	4.876	<0.001
***Ruminococcaceae***	**4**	**474**	**45.7**	**0.137**	**-1.948 (-2.168,-1.728)**	**-17.364**	**<0.001**
**Genus**							
*Faecalibacterium*	3	412	94.8	<0.001	3.496 (2.139,4.853)	5.050	<0.001
*Escherichia-Shigella*	5	498	95.0	<0.001	2.267 (1.155,3.378)	3.996	<0.001
*Megamonas*	4	474	93.5	<0.001	1.616 (0.784,2.447)	3.809	<0.001
***Prevotella***	**4**	**474**	**87.7**	**<0.001**	**-1.716 (-2.338,-1.095)**	**-5.413**	**<0.001**
*Roseburia*	5	517	98.2	<0.001	2.032 (-0.046,4.109)	1.916	0.055
***Ruminococcus***	**4**	**474**	**95.8**	**<0.001**	**-3.919 (-5.412,-2.426)**	**-5.144**	**<0.001**
*Bacillus*	3	412	87.1	<0.001	3.476 (2.618,4.335)	7.934	<0.001
*Blautia*	3	222	98.5	<0.001	2.143 (-1.114,5.401)	1.290	0.197

## 4 Discussion

Post-stroke depression is a common complication after a stroke. In addition to limb dysfunction, the clinical manifestations of patients are also accompanied by mental and emotional abnormalities, such as low mood, decreased interest in external things, and delayed thinking functions. These symptoms seriously reduce the compliance with medical activities of stroke survivors, which is not conducive to the recovery of limb function [27]. Studies have shown that long-term depression in patients has varying degrees of influence on their neurological, immune, and gastrointestinal functional states [28, 29, 30]. The effect of depression on gastrointestinal function is mainly reflected in the normal rhythmic movement of the gastrointestinal tract and the secretion of the mucosa, and the above two changes have a direct impact on the types and spatial distribution of intestinal flora [31]. Epidemiological research found that the main symptoms of gastrointestinal dysfunction in patients with post-stroke depression include: loss of appetite, functional constipation, abdominal distention, etc. [32], while some clinical symptoms were significantly correlated with the patient's psychobehavioral changes [33, 34]. With the proposal and gradual development of precision medicine in recent years, finding new targets closely related to the psychobehavioral changes in PSD patients, and implementing precision therapy has become the key to the treatment of this disease [35]. Growing evidence proposed that gut microbiota was disturbed in PSD patients, and the distinct microbiome characteristics might provide a novel target for PSD treatment.

In the present study, we conducted a meta-analysis and systematic review to assess gut microbiota perturbations in patients with PSD for the first time. The results revealed a discrepant abundance of intestinal bacterial phyla in PSD versus healthy individuals across the studies. However, no significant differences in the majority of richness and diversity indexes, except for observed species. PSD patients were observed with a higher observed species as compared to controls in a third of the studies. The differences in the abundance of the four major gut-dominant phyla are mainly as follows: *Firmicutes* were found to be dramatically lower in PSD versus controls, while the other three phyla (*Bacteroidetes*, *Proteobacteria*, and *Fusobacteria*) were revealed to be significantly lower in PSD versus controls.

Eight of the included studies investigated the gut microbiota using 16S rRNA-sequencing technology [17, 20, 21, 22, 23, 24, 25, 26], while the rest only detected three components of gut microbiota, including *Enterococcus faecalis, Bifidobacterium*, *Escherichia coli*, using the ATB expression identification system [16]. It found a significantly higher abundance of *Enterococcus faecalis* and *Escherichia coli*, as well as a significantly lower abundance of *Bifidobacterium* in PSD as compared to healthy controls. Despite unavailable data on these gut microbiota compositions in other high-throughout sequencing research, current studies may also suggest a possibly important role for these gut microbiota compositions in PSD patients. *Enterococcus faecalis* is a ubiquitous gram-positive bacterium common in the digestive tract and an important opportunistic pathogen [36]. In addition, *Escherichia coli* is a conditional pathogen that is commonly found in the intestines of humans and is associated with a variety of gastrointestinal infections [37]. The presence and abundance of *Bifidobacterium* have been indicated as a biomarker for health because *Bifidobacterium* confers a large range of health benefits to the metabolic and immune system. Antidepressant therapy targeting intestinal *Bifidobacterium* has also been developed [38]. In addition, this study also revealed the elevation of inflammatory factors in PSD and found significant correlations between gut microbiota and inflammatory factors. The elucidation of the causal relationship between gut microbiota disturbances and the aggravated inflammatory response would provide new clues for the development of anti-PSD drugs [39].

Six of eight studies conducting 16S rRNA-sequencing provided sufficient data for the pooled estimation of gut microbiota composition at the phylum level [17, 20, 22, 23, 24, 25, 26]. The pooled analysis of four dominant bacterial phyla generated consistent trends with that in depression patients [40]. Four articles reported that the relative abundance of *Bacteroidetes* in PSD was significantly higher than that in controls [22, 23, 24, 26], with an article reporting the opposite trend [20]. Meanwhile, the pooled estimate demonstrated a significantly lower relative abundance of *Firmicutes* in PSD than in controls. Despite insufficient data for the evaluation of the *Firmicutes/Bacteroidetes* ratio, we could infer its downtrend in the PSD based on the opposite trend of *Firmicutes* and *Bacteroidetes* in the PSD. The *Firmicutes* and *Bacteroidetes* are two kinds of predominant beneficial bacteria in the human gut with a stable ratio in healthy adults, whereas its disorder was closely related to obesity and diabetes [41, 42]. Zhang et al. found that the antidepressant effects of MR16-1, a mouse IL-6 receptor antibody, were closely associated with the improvement of the *Firmicutes/Bacteroidetes* ratio in mice [43]. *Proteobacteria* is the second largest group of bacteria, comprising several known human pathogens such as *Brucella* and *Rickettsia*. Accumulating evidence identified *Proteobacteria* as a possible signature of diseases, such as irritable bowel syndrome [44] and autoimmune thyroid disease [45]. The meta-analysis revealed a significantly higher *Proteobacteria* in PSD as compared to healthy controls. The existing hypothesis proposed that *Proteobacteria* bloom was associated with increased availability of oxygen resulting from the reduced beta-oxidative capacity of epithelial cells under stroke-induced intestinal inflammation [46]. Moreover, *Fusobacteria*, a small group of Gram-negative bacteria, was found to be significantly increased in PSD as compared to healthy individuals in this meta-analysis.

Further investigation revealed the dysregulation of various intestinal microbiota at the family and genus levels. Recently, it was reported that the intestinal content of *Acidaminococcaceae* was positively correlated with self-reported depression [47]. Consistent with this, our analysis showed a significantly elevated intestinal content of *Acidaminococcaceae* in PSD patients. In addition, we also observed significantly elevated content in the other three families and reduced content in five families. Three families including *Bacteroidaceae*, *Fusobacteriaceae*, and *Prevotellaceae* presented with the same change pattern in PSD and depression, suggesting their potential role in the occurrence and development of depression. Other families without similar change patterns may be closely related to stroke. For example, the modulation of *Lachnospiraceae* and *Ruminococcaceae* was suggested to contribute to the therapeutic effects of the combination of Puerariae Lobatae Radix and Chuanxiong Rhizoma on cerebral ischemic stroke [48]. Meanwhile, the enrichment of pathogens and opportunistic microorganisms, such as *Escherichia-Shigella* and *Faecalibacterium,* was responsible for the stroke-induced gut microbial disturbances [48]. These data revealed the similarities and differences between PSD and depression intestinal microbiota, indicating that depression induced by different diseases may have common and unique intestinal microbiota characteristics that have potential value in disease diagnosis and drug development.

### 4.1 Limitations

There are several limitations in this meta-analysis. Firstly, the sample size of included studies is small, which might make the results less robust. Secondly, part of the analysis data was indirectly extracted from the article, which may deviate from the real data. Finally, due to the unavailable high-throughout sequencing data, we failed to conduct an individual-based meta-analysis using a standardized bioinformatic pipeline that could help to clarify the discrepant findings.

## 5 Conclusion

In the present study, we revealed discrepant distribution in the intestinal flora of PSD patients versus healthy controls at the phylum, family and genus levels. Higher observed species in PSD versus controls were observed, whereas the Good's coverage, Chao1, ACE, evenness, and alpha diversity indexes yielded no significant differences. However, these findings are not sufficient to generalize to a larger population given the small number of studies and sample size as well as substantial heterogeneity. Further high-quality studies are required to validate these findings. Meanwhile, an individual-level meta-analysis with standardized analysis pipelines should be developed and adopted to exclude the differences in the generation and processing of 16S rRNA-sequencing data.

## Declarations

### Author contribution statement

Fang Luo and Chengbing Fang: Conceived and designed the experiments; Performed the experiments; Analyzed and interpreted the data; Contributed reagents, materials, analysis tools or data; Wrote the paper.

### Funding statement

This research did not receive any specific grant from funding agencies in the public, commercial, or not-for-profit sectors.

### Data availability statement

Data will be made available on request.

### Declaration of interests statement

The authors declare no competing interests.

### Additional information

No additional information is available for this paper.
